# Impact of prediabetes and diabetes on 3-year outcome of patients treated with new-generation drug-eluting stents in two large-scale randomized clinical trials

**DOI:** 10.1186/s12933-021-01405-4

**Published:** 2021-10-30

**Authors:** Eline H. Ploumen, Tineke H. Pinxterhuis, Paolo Zocca, Ariel Roguin, Rutger L. Anthonio, Carl E. Schotborgh, Edouard Benit, Adel Aminian, Peter W. Danse, Carine J. M. Doggen, Clemens von Birgelen, Marlies M. Kok

**Affiliations:** 1 Department of Cardiology, Thoraxcentrum Twente, Medisch Spectrum Twente, Koningsplein 1, 7512 KZ Enschede, The Netherlands; 2 Department of Health Technology and Services Research, Faculty of Behavioural, Management and Social Sciences, Technical Medical Centre, University of Twente, Enschede, Netherlands; 3 Department of Cardiology, Hadera and B. Rappaport-Faculty of Medicine, Hillel Yaffe Medical Center, Israel Institute of Technology, Haifa, Israel; 4 Department of Cardiology, Treant Zorggroep, Scheper Hospital, Emmen, Netherlands; 5 Department of Cardiology, Haga Hospital, The Hague, Netherlands; 6 Department of Cardiology, Jessa Hospital, Hasselt, Belgium; 7 Department of Cardiology, Centre Hospitalier Universitaire de Charleroi, Charleroi, Belgium; 8 Department of Cardiology, Rijnstate Hospital, Arnhem, Netherlands

**Keywords:** Diabetes mellitus, Prediabetes, Drug-eluting stents, Percutaneous coronary intervention, Coronary artery disease, Randomized clinical trial

## Abstract

**Background:**

Diabetes is associated with adverse outcomes after percutaneous coronary intervention with drug-eluting stents (DES), but for prediabetes this association has not been definitely established. Furthermore, in patients with prediabetes treated with contemporary stents, bleeding data are lacking. We assessed 3-year ischemic and bleeding outcomes following treatment with new-generation DES in patients with prediabetes and diabetes as compared to normoglycemia.

**Methods:**

For this post-hoc analysis, we pooled patient-level data of the BIO-RESORT and BIONYX stent trials which both stratified for diabetes at randomization. Both trials were multicenter studies performed in tertiary cardiac centers. Study participants were patients of whom glycemic state was known based on hemoglobin A1c, fasting plasma glucose, or medically treated diabetes. Three-year follow-up was available in 4212/4330 (97.3 %) patients. The main endpoint was target vessel failure, a composite of cardiac death, target vessel myocardial infarction, or target vessel revascularization.

**Results:**

Baseline cardiovascular risk profiles were progressively abnormal in patients with normoglycemia, prediabetes, and diabetes. The main endpoint occurred in 54/489 patients with prediabetes (11.2 %) and 197/1488 with diabetes (13.7 %), as compared to 142/2,353 with normoglycemia (6.1 %) (HR: 1.89, 95 %-CI 1.38–2.58, p < 0.001, and HR: 2.30, 95 %-CI 1.85–2.86, p < 0.001, respectively). In patients with prediabetes, cardiac death and target vessel revascularization rates were significantly higher (HR: 2.81, 95 %-CI 1.49–5.30, p = 0.001, and HR: 1.92, 95 %-CI 1.29–2.87, p = 0.001), and in patients with diabetes all individual components of the main endpoint were significantly higher than in patients with normoglycemia (all p ≤ 0.001). Results were consistent after adjustment for confounders. Major bleeding rates were significantly higher in patients with prediabetes and diabetes, as compared to normoglycemia (3.9 % and 4.1 % vs. 2.3 %; HR:1.73, 95 %-CI 1.03–2.92, p = 0.040, and HR:1.78, 95 %-CI 1.23–2.57, p = 0.002). However, after adjustment for confounders, differences were no longer significant.

**Conclusions:**

Not only patients with diabetes but also patients with prediabetes represent a high-risk population. After treatment with new-generation DES, both patient groups had higher risks of ischemic and bleeding events. Differences in major bleeding were mainly attributable to dissimilarities in baseline characteristics. Routine assessment of glycemic state may help to identify patients with prediabetes for intensified management of cardiovascular risk factors.

*Trial registration*: BIO-RESORT ClinicalTrials.gov: NCT01674803, registered 29-08-2012; BIONYX ClinicalTrials.gov: NCT02508714, registered 27-7-2015.

**Supplementary Information:**

The online version contains supplementary material available at 10.1186/s12933-021-01405-4.

## Background

The presence of diabetes is a well-known risk factor for coronary artery disease and has been associated with an increased adverse event risk after percutaneous coronary intervention (PCI) with drug-eluting stents (DES) [1–5]. Refinements in stent technology and concomitant medical therapy have improved outcomes, yet, diabetic patients still show higher adverse event rates [1–5]. The increased risk of ischemic events has been linked to the presence of a prothrombotic state due to platelet hyperactivity, increased platelet aggregation, and endothelial dysfunction [6–8]. While ischemic outcomes of diabetic patients following DES implantation have been evaluated, such data for patients with *pre*diabetes are scarce. Furthermore, conflicting data have been reported regarding bleeding risk in diabetic patients undergoing PCI [9–11], and there is a lack of data on bleeding in patients with prediabetes.

BIO-RESORT and BIONYX, two large-scale randomized clinical trials in all-comer patients undergoing PCI for obstructive coronary artery disease, have established non-inferiority of several new-generation DES versus contemporary reference DES [12, 13]. Furthermore, in patients with diabetes no difference in outcome was seen between DES groups [14]. For the present analysis at 3 years, we examined pooled patient-level data of trial participants with known glycemic state, based on hemoglobin A1c (HbA1c) and/or fasting plasma glucose (FPG) testing, or medically treated diabetes. Subsequently, we assessed potential differences in the incidence of ischemic and bleeding events after PCI with new-generation DES in patients with prediabetes and diabetes as compared to normoglycemia.

## Methods

### Study design and participants

For the current analysis, we pooled patient-level data of two randomized trials of which design and details have been published [12, 13]. In brief, BIO-RESORT (Comparison of biodegradable polymer and durable polymer drug-eluting stents in an all-comers population; ClinicalTrials.gov *NCT01674803*) is a 3-arm, patient- and assessor-blinded trial, performed at four cardiac centers in the Netherlands. From December 2012 to August 2015, patients were randomized (1:1:1) to treatment with the ultrathin strut biodegradable polymer sirolimus-eluting Orsiro stent (Biotronik, Bülach, Switzerland), the very thin strut biodegradable polymer everolimus-eluting Synergy stent (Boston Scientific, Marlborough, MA) or the thin strut durable polymer zotarolimus-eluting Resolute Integrity stent (Medtronic, Santa Rosa, CA) [12]. BIONYX (Bioresorbable polymer-coated Orsiro versus durable polymer-coated Resolute ONYX stents; ClinicalTrials.gov *NCT025087140*) is a patient- and assessor-blinded trial that was performed in seven cardiac centers in Israel, Belgium, and the Netherlands. From October 2015 to December 2016, patients were randomized (1:1) to treatment with the thin strut durable polymer zotarolimus-eluting Resolute Onyx stent (Medtronic) or the ultrathin strut biodegradable polymer sirolimus-eluting stent 13.

**Fig. 1:**
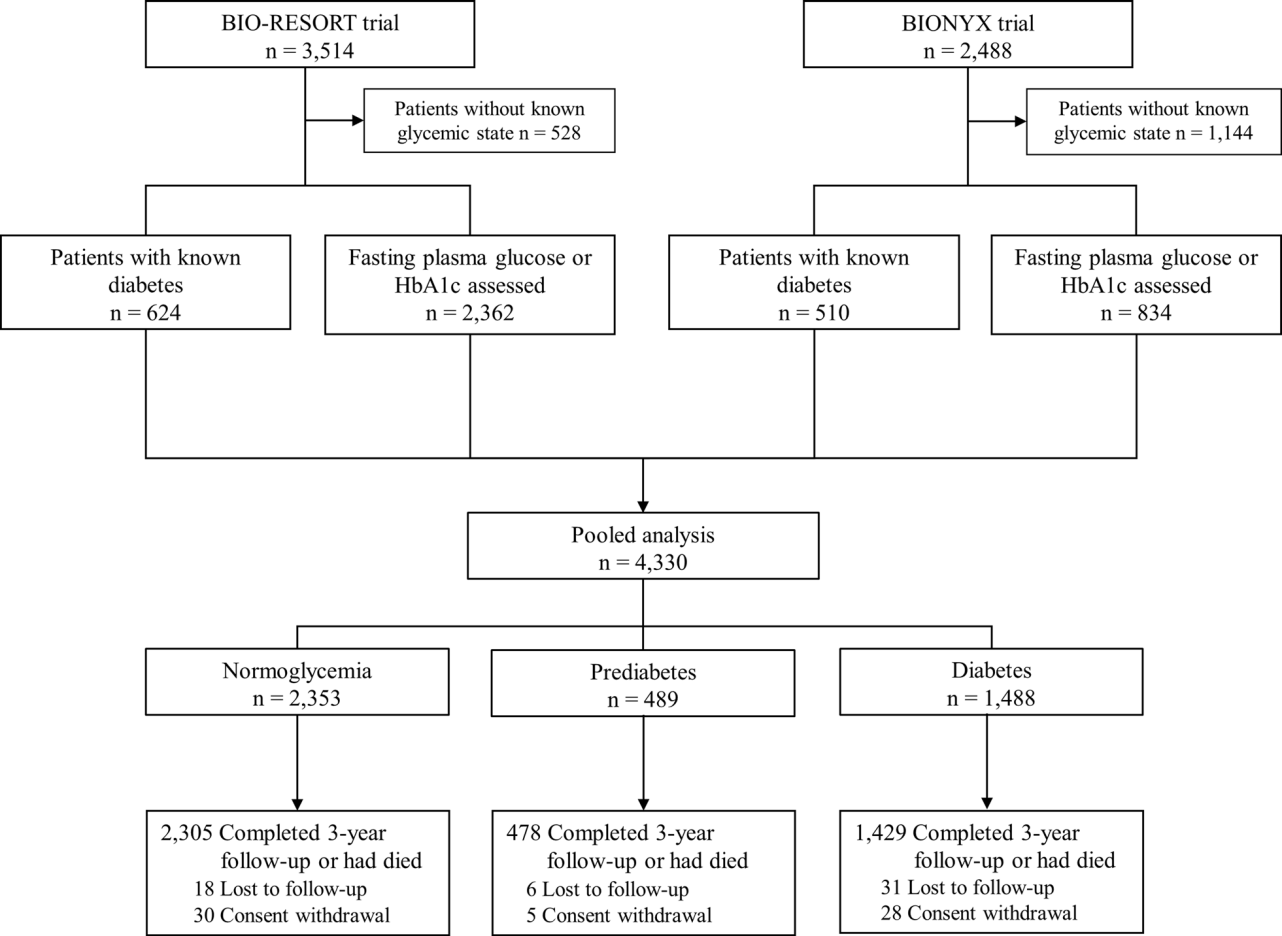
Study flow diagram. Study flow diagram showing the number of patients included from each randomized clinical trial

For both trials, patients were eligible for enrollment if they were 18 years or older, capable of providing informed consent, and required PCI. Exclusion criteria were very limited, and included intolerance to dual antiplatelet therapy (DAPT), known pregnancy, and life expectancy of < 1 year. There was no restriction for clinical syndrome, target lesion type, lesion length, reference vessel size, and number of lesions or vessels to be treated. Web-based randomization was performed with the use of a custom designed computer program in random block sizes of 6 and 3, stratified according to the presence of diabetes mellitus. Follow-up will be extended up to 5-years. Figure 1 shows the study flow diagram. The trials complied with the Declaration of Helsinki and were approved by the Medical Ethics Committee Twente and the Institutional Review Boards of all centers. All patients provided written informed consent.

### Procedures, follow-up, and monitoring

All coronary interventions were performed according to international medical guidelines and the operator’s judgment. Detailed information on the contemporary DES that were used have been published [12, 13]. DAPT was generally prescribed for 12 months in patients with acute coronary syndromes (ACS) and for 6 months in patients with stable angina, as guidelines recommended during the study periods. All study sites were encouraged to measure FPG and HbA1c shortly before or after index procedure. Clinical follow-up was obtained at visits to the outpatient clinic, by telephone, or by paper-based questionnaire. The trials were monitored (Diagram, Zwolle, Netherlands), and events were adjudicated by independent committees that were blinded for treatment (Diagram, Zwolle, the Netherlands, or a committee of cardiologists of University of Amsterdam, The Netherlands).

### Clinical endpoints and definitions

Clinical endpoints were prespecified according to the Academic Research Consortium [15, 16]. The main endpoint was target vessel failure (TVF), a composite of safety and efficacy, consisting of cardiac death, target vessel-related myocardial infarction (MI), or clinically indicated target vessel revascularization. Secondary composite endpoints included target lesion failure (cardiac death, target vessel MI, or clinically indicated target lesion revascularization) and major adverse cardiac events (all-cause death, any MI, or clinically-indicated target lesion revascularization). Other secondary endpoints were all-cause mortality, the individual components of TVF, target lesion revascularization, bleeding, and both definite and definite-or-probable stent thrombosis. The endpoint any MI included peri-procedural MI and was based on definitions from the Academic Research Consortium [16]. Major bleeding was defined as class 3 or 5 of the Bleeding Academic Research Consortium (BARC 3a, 3b, 3c, 5a, 5b) and/or all Thrombolysis in Myocardial Infarction (TIMI) major bleedings [17, 18].

Definitions of glycemic state were based on the World Health Organization definition and diagnosis of diabetes mellitus and intermediate hyperglycemia statement [19] and the International Expert Committee 2009 criteria for HbA1c with FPG [20]. Normoglycemia was defined as FPG < 6.1 mmol/l and/or HbA1c ≤ 41 mmol/mol, prediabetes as FPG 6.1–6.9mmol/l and/or HbA1c 42–47 mmol/mol, and diabetes as FPG ≥ 7.0 mmol/l and/or HbA1c ≥ 48 mmol/mol.

### Statistical analysis

Differences in categorical variables were examined with Pearson’s χ^2^ or Fisher’s exact test, and differences in continuous variables with Mann-Whitney *U* or *t* test, as appropriate. Kaplan–Meier methods were used to assess time-to-endpoints. Hazard ratios (HR) with 2-sided confidence intervals (CI) were computed by Cox proportional hazards analysis. A two-sided p-value < 0.05 was considered significant. All analyses were performed according to the intention-to-treat principle. A multivariable model was constructed, including all baseline variables that showed a between-group difference and an association with TVF (p < 0.15). The final model was made using step-wise backward selection. It included: clinical presentation; number of diseased vessels; at least one severely calcified lesion treated. In the same manner, a multivariable model was constructed for bleeding endpoints. Based on stepwise backward selection, age, sex, hypercholesterolemia, vitamin K antagonist use at 3-year follow-up, and multivessel treatment were included, and based on literature, hypertension, renal insufficiency, and previous stroke. Statistical analyses were done with SPSS version-24.0 (IBM, Armonk, NY).

## Results

Three-year follow-up was available in 4212/4330 (97.3 %) patients. Consent withdrawal was balanced between groups (n = 63). Slightly more patients with diabetes were lost to follow-up: normoglycemia 0.8 %, prediabetes 1.3 %, diabetes 2.2 % (total n = 55). As may be expected, baseline patient, lesion, and procedural characteristics showed significant between-group differences (Table 1). Baseline cardiovascular risk profiles were progressively abnormal in patients with normoglycemia, prediabetes, and diabetes, with the exception of smoking which was more common among patients with normoglycemia. In addition, patients with prediabetes or diabetes had smaller vessels, more severely calcified lesions and more bypass grafts treated. At 3-years, DAPT was used by 5.7 % of patients with normoglycemia, 9.2 % with prediabetes, and 12.1 % with diabetes (p < 0.001; Additional file 1: Table S1). Vitamin K antagonist use differed across groups (normoglycemia 7.5 %, prediabetes 10.0 %, diabetes 10.7 %; p = 0.003), while direct oral anticoagulant use was similar (5.2 %, 6.8 %, 5.9 %, respectively; p = 0.35).

**Table 1:** Baseline patient, lesion and procedural characteristics

	Baseline characteristics, No. (%)			
	Normoglycemia n = 2353	Prediabetes n = 489	Diabetes n = 1488	P-value
Age, mean (SD), years	62.9 (10.8)	64.6 (10.8)	65.7 (10.6)	< 0.001
Female	568 (24.1)	142 (29.0)	439 (29.5)	< 0.001
Body mass index, mean (SD), kg/m^2^	26.9 (3.8)	27.9 (4.3)	29.1 (4.7)	< 0.001
Hypertension	933 (39.7)	236/488 (48.4)	948/1484 (63.9)	< 0.001
Hypercholesterolemia	909/2351 (38.7)	204/488 (41.8)	746/1479 (50.4)	< 0.001
Current smoker	764/2299 (33.2)	140/467 (30.0)	376/1440 (26.1)	< 0.001
Renal insufficiency^a^	55 (2.3)	21 (4.3)	114 (7.7)	< 0.001
Previous stroke	137 (5.8)	35 (7.2)	137 (9.2)	< 0.001
Previous myocardial infarction	350 (14.9)	91 (18.6)	320 (21.5)	< 0.001
Previous PCI	359 (15.3)	100 (20.4)	368 (24.7)	< 0.001
Previous CABG	140 (5.9)	40 (8.2)	156 (10.5)	< 0.001
Clinical presentation				0.001
STEMI	763 (32.4)	146 (29.9)	379 (25.5)	
Non-STEMI	521 (22.1)	98 (20.0)	345 (23.2)	
Unstable angina	389 (16.5)	94 (19.2)	278 (18.7)	
Stable angina	680 (28.9)	151 (30.9)	486 (32.7)	
Lesion and procedural characteristics				
At least 1 severely calcified lesion	450 (19.1)	108 (22.1)	344 (23.1)	0.009
At least 1 bifurcation treated	828 (35.2)	183 (37.4)	551 (37.0)	0.41
Graft treated	32 (1.4)	12 (2.5)	37 (2.5)	0.026
Small vessel treated (< 2.75mm)	1256 (53.4)	282 (57.7)	910 (61.2)	< 0.001
Multivessel treatment	398 (16.9)	100 (20.4)	275 (18.5)	0.13
Total stent length/patient, median (IQR)	32 (20–52)	31 (20–53)	30 (18–48)	0.86
Direct stenting	265 (15.6)	53 (14.1)	147 (16.1)	0.67
Postdilation	1913 (81.3)	399 (81.6)	1,121(75.3)	< 0.001
Number of diseased vessels > 50 % angiographic stenosis				< 0.001
One vessel disease	1380 (58.6)	274 (56.0)	757 (50.9)	
Two vessel disease	737 (31.3)	158 (32.3)	487 (32.7)	
Three vessel disease	236 (10.0)	57 (11.7)	244 (16.4)	

Values are n/N (%), mean (SD), or median (IQR)

CABG, coronary artery bypass grafting; PCI, percutaneous coronary intervention; STEMI, ST-segment-elevation myocardial infarction; NSTEMI, non-ST-segment-elevation myocardial infarction

^a^Defined as creatinine level ≥ 130 µmol/l, an estimated glomerular filtration rate of less than 30 ml per minute per 1.73 m^2^ of body-surface area, or the need for dialysis

### Prediabetes versus normoglycemia

**Fig. 2:**
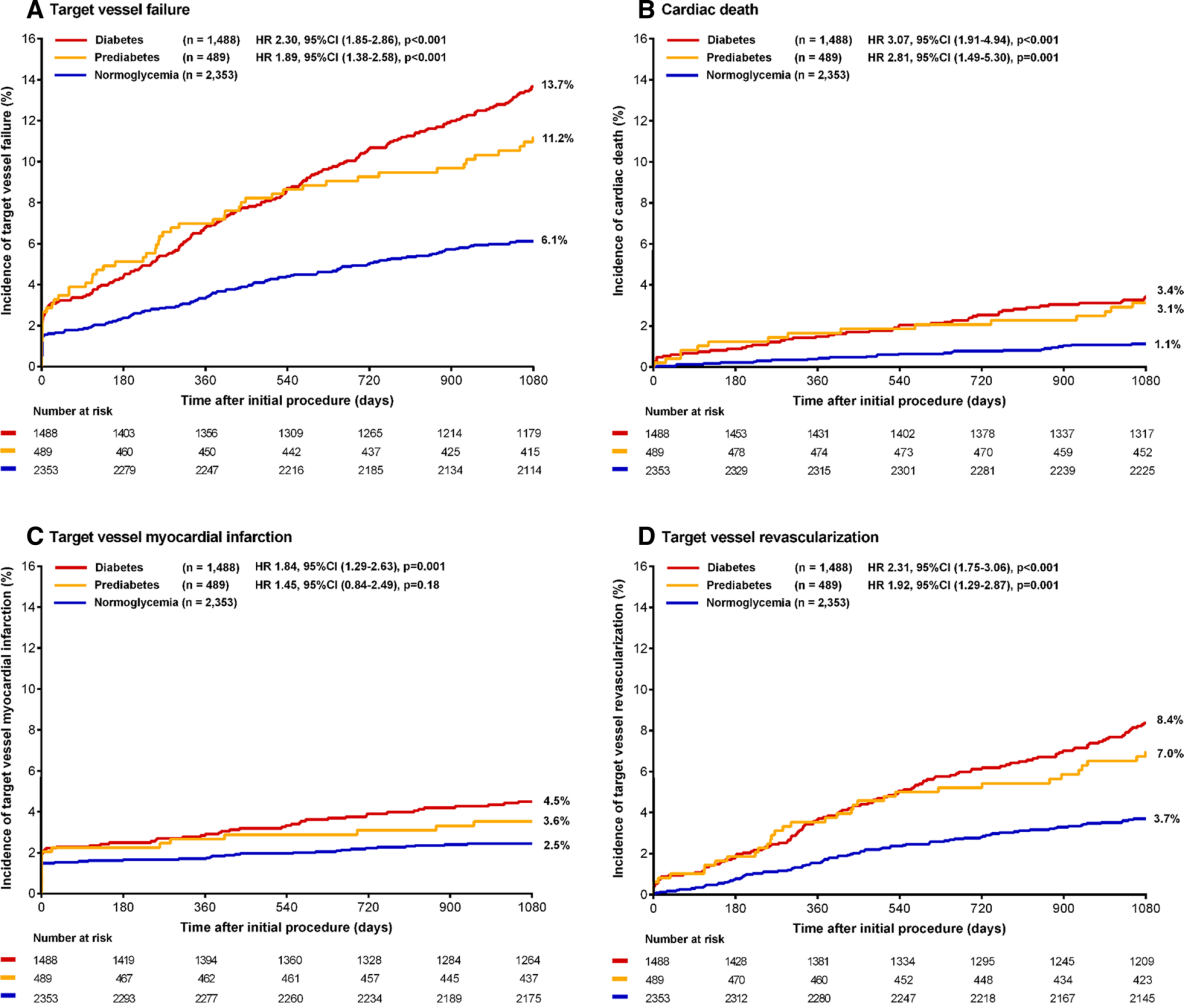
Kaplan–Meier event curves for target vessel failure and its individual components at 3-year follow-up. Kaplan–Meier event curves for target vessel failure and its individual components up to 3-year follow-up showing higher rates of the main endpoint in patients with prediabetes and diabetes as compared to patients with normoglycemia

**Fig. 3:**
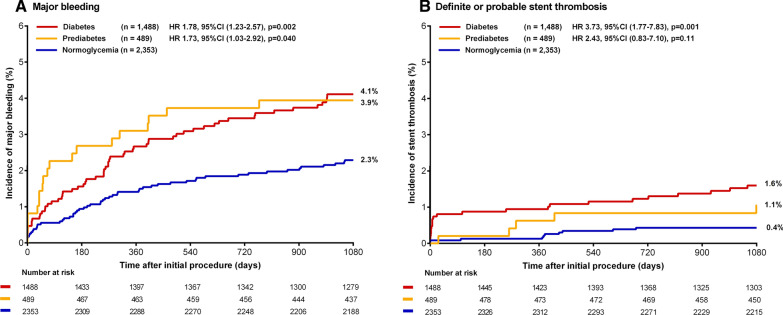
Kaplan–Meier event curves for major bleeding and stent thrombosis at 3-year follow-up. Kaplan–Meier event curves for major bleeding and stent thrombosis up to 3-year follow-up showing higher rates of major bleeding in patients with prediabetes and diabetes, and higher rates of definite-or-probable stent thrombosis in patients with diabetes as compared to patients with normoglycemia

The 3-year rate of the main composite endpoint TVF was significantly higher in patients with prediabetes as compared to patients with normoglycemia (11.2 % vs.6.1 %, HR: 1.89, 95 %-CI 1.38–2.58, p < 0.001) (Table 2). The rates of cardiac death and target vessel revascularization were also higher in patients with prediabetes (3.1 % vs. 1.1 %, HR: 2.81, 95 %-CI 1.49–5.30, p = 0.001, and 7.0 % vs. 3.7 %, HR: 1.92, 95 %-CI 1.29–2.87, p = 0.001). Target vessel MI did not differ between groups (3.6 % vs. 2.5 %, HR: 1.45, 95 %-CI 0.84–2.49, p = 0.18). Figure 2 displays Kaplan–Meier event curves for TVF and components. All-cause mortality was higher in patients with prediabetes (5.4 % vs. 3.4 %, HR:1.59, 95 %-CI 1.02–2.47, p = 0.041). There was no significant between-group difference in definite stent thrombosis (HR: 1.08, 95 %-CI 0.23–4.99, p = 0.92). The rate of any bleeding did not differ between groups (5.9 % vs. 4.2 %; HR: 1.43, 95 %-CI 0.94–2.18, p = 0.09), while the major bleeding rate was higher in patients with prediabetes (3.9 % vs. 2.3 %, HR: 1.73, 95 %-CI 1.03–2.92, p = 0.040; Fig. 3). The incidence of TVF was significantly higher in patients with prediabetes, irrespective of whether patients presented with ACS at index procedure (Additional file 1: Table S2). Among patients with ACS, the incidence of major bleeding was higher in the presence of prediabetes, while among patients with stable angina the major bleeding rate was similar in both patients with prediabetes and normoglycemia (Additional file 1: Table S2).

**Table 2:** Three-year clinical outcome of patients with normoglycemia, prediabetes or diabetes

	Normo-glycemia n=2353	Prediabetes n=489	Diabetes n=1488	Prediabetes vs. Normoglycemia				Diabetes vs. Normoglycemia			
				Unadjusted		Adjusted		Unadjusted		Adjusted	
				HR (95 % CI)	p-value	HR (95 %CI)	p-value	HR (95 % CI)	p-value	HR (95 % CI)	p-value
Target vessel failure	142 (6.1)	54 (11.2)	197 (13.7)	1.89 (1.38–2.58)	< 0.001	1.79 (1.31–2.45)	< 0.001	2.30 (1.85–2.86)	< 0.001	2.03 (1.63–2.52)	< 0.001
Any death	80 (3.4)	26 (5.4)	112 (7.7)	1.59 (1.02–2.47)	0.04	1.54 (0.99–2.40)	0.06	2.3 (1.72–3.05)	< 0.001	2.11 (1.58–2.82)	< 0.001
Cardiac death	26 (1.1)	15 (3.1)	49 (3.4)	2.81 (1.49–5.30)	0.001	2.70 (1.43–5.10)	0.002	3.1 (1.91–4.94)	< 0.001	2.76 (1.71–4.45)	< 0.001
Any MI	72 (3.1)	23 (4.8)	87 (6.1)	1.56 (0.98–2.49)	0.06	1.49 (0.93–2.38)	0.10	2.0 (1.45–2.70)	< 0.001	1.77 (1.29–2.42)	< 0.001
Target vessel MI	57 (2.5)	17 (3.6)	65 (4.5)	1.45 (0.84–2.49)	0.18	1.36 (0.79–2.33)	0.27	1.84 (1.29–2.63)	0.001	1.59 (1.11–2.28)	0.011
Any revascularization	159 (6.9)	55 (11.6)	195 (13.8)	1.72 (1.27–2.34)	0.001	1.66 (1.22–2.25)	0.001	2.07 (1.68–2.56)	< 0.001	1.88 (1.52–2.32)	< 0.001
Target vessel revascularization	85 (3.7)	33 (7.0)	118 (8.4)	1.92 (1.29–2.87)	0.001	1.81 (1.21–2.71)	0.004	2.31 (1.75–3.06)	< 0.001	2.05 (1.55–2.71)	< 0.001
Target lesion revascularization	49 (2.1)	26 (5.5)	78 (5.5)	2.62 (1.63–4.22)	< 0.001	2.48 (1.54–4.00)	< 0.001	2.63 (1.84–3.76)	< 0.001	2.33 (1.62–3.34)	< 0.001
Target lesion failure	115 (5.0)	49 (10.2)	159 (11.0)	2.11 (1.51–2.94)	< 0.001	2.01 (1.44–2.80)	< 0.001	2.28 (1.79–2.89)	< 0.001	2.00 (1.57–2.55)	< 0.001
MACE	179 (7.7)	63 (13.0)	235 (16.0)	1.75 (1.31–2.33)	< 0.001	1.69 (1.27–2.25)	< 0.001	2.19 (1.80–2.66)	< 0.001	1.98 (1.62–2.40)	< 0.001
Definite-or-probable stent thrombosis	10 (0.4)	5 (1.1)	23 (1.6)	2.43 (0.83–7.10)	0.11	2.30 (0.79–6.75)	0.13	3.73 (1.77–7.83)	0.001	3.35 (1.58–7.09)	0.002
Definite stent thrombosis	9 (0.4)	2 (0.4)	14 (1.0)	1.08 (0.23–4.99)	0.92	1.03 (0.22–4.78)	0.97	2.52 (1.09–5.82)	0.030	2.30 (0.99–5.37)	0.053
Bleeding	96 (4.2)	28 (5.9)	93 (6.5)	1.43 (0.94–2.18)	0.09	1.32 (0.83–2.09)	0.24	1.59 (1.19–2.11)	0.001	1.19 (0.85–1.67)	0.32
Major Bleeding	53 (2.3)	19 (3.9)	59 (4.1)	1.76 (1.04–3.98)	0.034	1.60 (0.89–2.91)	0.12	1.81(1.25–2.63)	0.002	1.35 (0.89–2.12)	0.20

Values are n (%)

MI, myocardial infarction; MACE, major adverse cardiac events

After adjustment for confounders, multivariable analysis still showed a significantly higher TVF risk for patients with prediabetes as compared to patients with normoglycemia (Table 2). This was also the case for cardiac death and target vessel revascularization, while all-cause mortality risk was no longer significantly higher. The risk of any bleeding and major bleeding showed no independent association with prediabetes (Table 2).

### Diabetes versus normoglycemia

Patients with diabetes, as compared to patients with normoglycemia, had significantly higher rates of TVF (13.7 % vs. 6.1 %, HR:2.30, 95 %-CI 1.85–2.86, p <  0.001) and its components (Table 2). Figure 2 displays Kaplan–Meier curves for TVF and its components. The incidence of TVF was significantly higher in patients with diabetes, irrespective of whether they presented with ACS at index procedure (Additional file 1: Table S2). Furthermore, definite stent thrombosis occurred more frequently in patients with diabetes (1.0 % vs.0.4 %, HR:2.52, 95 %-CI 1.09–5.82, p = 0.030). The rates of other individual and composite endpoints were also significantly higher in patients with diabetes (Table 2), including any bleeding (6.5 % vs. 4.2 %, HR:1.59, 95 %-CI 1.19–2.11, p = 0.001), as well as major bleeding (4.1 % vs. 2.3 %, HR: 1.78, 95 %-CI 1.23–2.57, p = 0.002; Fig. 3). When separately assessing patients with acute and stable coronary syndromes, patients with diabetes showed a higher major bleeding rate only in the ACS but not in stable coronary syndrome group (Table S2).

Multivariable analysis showed that the risk of TVF remained significantly higher for patients with diabetes as compared to patients with normoglycemia (Table 2). Similarly, patients with diabetes had significantly higher risks of other endpoints. Yet, the risk of any and major bleeding showed no independent association with diabetes.

## Discussion

This analysis of pooled patient-level data from two large-scale randomized clinical trials which assessed new-generation DES, showed that not only patients with diabetes but also patients with prediabetes represent a high-risk PCI population. There was a higher 3-year rate of the main safety and efficacy endpoint for both patient groups as compared to patients with normoglycemia. In patients with prediabetes, this difference was driven by cardiac death and target vessel revascularization, while in patients with diabetes it was also based on target vessel-related MI.

In patients with prediabetes, only the risks of repeated revascularization and cardiac death remained significantly higher after adjustment for confounders. Patients with diabetes showed higher risks of all ischemic clinical endpoints, including definite-or-probable stent thrombosis, which was consistent after adjustment. Furthermore, the major bleeding risk was higher in both, patients with prediabetes and diabetes. However, after adjustment for the potential confounders the two glycemic states were not independently associated with major bleeding. In other words, the higher bleeding risk in patients with prediabetes and diabetes can largely be explained by comorbidities and demographics that are related to an increased bleeding risk.

### Ischemic outcomes

Several studies assessed patients with diabetes who were treated with new-generation DES, and showed a higher risk of adverse events following PCI, including ischemic outcomes [4, 5, 21, 22]. Yet, data on PCI patients with prediabetes are scarce. The South Korean KAMIR registry examined patients with acute MI treated with contemporary DES, and compared 3709 patients with prediabetes and 5173 patients with diabetes to 3080 patients with normoglycemia [23]. The findings of the present analysis corroborate that study which showed in patients with prediabetes and diabetes higher 2-year rates of revascularization and cardiac death, and in patients with diabetes also higher rates of all-cause mortality and MI. A previous subgroup analysis of BIO-RESORT assessed 324 patients with prediabetes, 793 with diabetes, and 1869 with normoglycemia and showed that patients with prediabetes, similar to patients with diabetes, had higher 1-year risks of mortality and repeat revascularization after treatment with contemporary DES [4]. Furthermore, the BIO-RESORT Silent Diabetes study [24] previously reported that patients with prediabetes and silent diabetes—assessed by oral glucose tolerance testing and HbA1c at the time of the index procedure—had a higher 3-year risk of TVF. Yet, this was mainly driven by events during the first 48 h. Those previously reported data and the findings of the present study suggest that patients with prediabetes have an increased risk of ischemic events. Therefore, patients with prediabetes should be considered high-risk, and routine assessment of glycemic state in PCI patients may help to target this group for intensified management of cardiovascular risk factors.

### Bleeding outcomes

While in the current analysis patients with prediabetes and diabetes experienced more ischemic events, both groups also showed higher rates of major bleeding (Fig. 3). At baseline, patients with diabetes had higher rates of several risk factors for bleeding, such as hypertension, renal insufficiency, previous stroke, and older age. In addition, at 3-year follow-up they more often used anticoagulant therapy. After adjustment for such potential confounders, the differences in major bleeding were no longer significant.

So far, bleeding risk in patients with prediabetes treated with DES had not been assessed. Previous studies that assessed bleeding in diabetic patients showed conflicting results. In the randomized GLOBAL LEADERS trial, long-term ticagrelor monotherapy (after 1-month DAPT) was compared to conventional DAPT in 15,968 all-comer patients who underwent PCI with DES [10]. The study found no statistically significant difference in major bleeding in 4038 patients with diabetes as compared to patients without diabetes. Another study that evaluated DAPT strategies and examined bleeding risk was PLATO which assessed DAPT with ticagrelor versus clopidogrel in 11,289 patients with acute coronary syndrome, irrespective of treatment strategy (conservative treatment included). A substudy [9] in diabetic patients showed a higher rate of major bleeding in 2520 patients with diabetes, which was still apparent after adjustment for confounders. Considering all of the above, it is questionable whether hyperglycemia on its own increases bleeding risk, but it is quite clear that the cardiovascular risk profiles of patients with diabetes and prediabetes do increase that risk.

### DAPT strategies

The balance between ischemic and bleeding events in patients with diabetes is delicate, and can be challenging to manage. Several studies have investigated alternative antiplatelet strategies for these patients. The THEMIS trial assessed 19,220 patients with diabetes and stable coronary artery disease, who were randomized to treatment with aspirin only or DAPT with ticagrelor [25]. The patients on DAPT had lower rates of ischemic events, but this was offset by an increase in major bleedings. In our study, diabetic patients treated for stable angina did not show a higher incidence of major bleeding than patients with normoglycemia. Yet, this may be related to the modest sample size of the subgroup with stable angina. A meta-analysis that compared short-term (≤ 6 months) and long-term (12 months) DAPT following PCI in patients with diabetes (40 % ACS) found no difference in major adverse cardiac events, but a higher rate of major bleeding in patients on long-term DAPT [21]. However, in patients with diabetes, but not in those without diabetes, the definite-or-probable stent thrombosis rate was lower after long-term DAPT. In 7119 high-risk PCI patients, the TWILIGHT study assessed ticagrelor monotherapy following 3 months of DAPT versus conventional DAPT and found in the ticagrelor monotherapy group a lower incidence of bleeding without increase in ischemic events [26]. These results were consistent in patients with diabetes. These findings are certainly promising, yet the optimal DAPT strategy for patients with diabetes (or prediabetes) is a matter of ongoing discussion and warrants further research.

### Stent thrombosis

In patients with prediabetes and normoglycemia, definite-or-probable stent thrombosis occurred at comparable rates, but in patients with diabetes it occurred more often. While findings of such infrequent events are no more than hypothesis generating, it may be of interest that a similar between-group distribution of stent thromboses was seen in the acute MI patients of the KAMIR registry. That study also found a higher 2-year incidence of stent thromboses in patients with diabetes (1.0 %), but not with prediabetes (0.6 %), as compared to patients with normoglycemia (0.5 %) [23]. A meta-analysis that assessed stent thrombosis following PCI with DES showed that 5123 patients with diabetes had a higher late stent thrombosis rate than 13,775 normoglycemic patients [27]. The increased stent thrombosis risk in patients with diabetes may in part be related to their hypercoagulable state. The prothrombotic setting is promoted via several pathways, such as platelet hyperactivity, increased platelet aggregation, endothelial dysfunction, and elevated levels of multiple clotting factors [6–8]. Another mechanism that may increase stent thrombosis risk in diabetic patients is a reduction of early arterial healing, which leads to more uncovered stent struts [28] that were found to be associated with coronary stent thrombosis [29]. Furthermore, stent sizing and apposition may be suboptimal in patients with diabetes due to more diffuse and calcified coronary artery disease. If there is diffuse coronary artery disease, true vessel dimensions may be underestimated, as ‘reference segments’ may also be diseased. Suboptimal stent sizing and apposition, prothrombotic setting, and delayed arterial healing may all contribute to the increased stent thrombosis risk of patients with diabetes, which in the **present study was most pronounced during the acute and subacute phase. The risk of MI was also higher in patients with diabetes. As discussed above, there are several factors that increase the risk of stent thrombosis in diabetic patients, and these factors are also relevant to the occurrence of MI. Furthermore, in patients with diabetes who may have a higher plaque burden, the risk of** micro-embolization of atherothrombotic debris is increased which can lead to peri-procedural MI.

### Strengths and limitations

The present study analyzed patient-level pooled data of two large-scale randomized trials that primarily evaluated PCI with new-generation DES and stratified for diabetes at randomization. We examined 4330 patients in whom information on their glycemic state was available. Both trials applied the same definitions of baseline characteristics and clinical endpoints, assessed a relatively long follow-up, underwent independent monitoring, and reported clinical events following assessment by independent clinical event committees. In addition, for a PCI study, the groups of patients with diabetes and prediabetes are sizable. Nevertheless, the study also has several limitations. This analysis was not prespecified and not powered to provide definite conclusions regarding subpopulations with (pre)diabetes. Therefore, the findings are hypothesis generating. Furthermore, we cannot exclude unmeasured confounders. Information on the use of antidiabetic medication and glycemic control during follow-up is not available. Therefore, we do not know how many patients with prediabetes progressed to diabetes during follow-up, and it is unknown whether good or poor metabolic control affected the outcome of patients. Yet, the purpose of this study was to evaluate whether information on the glycemic state at the time of index PCI can be used to assess ischemic and bleeding risks of patients with (pre)diabetes in order to identify a high-risk group. We used four different new-generation DES, which theoretically could have affected clinical outcome. However, stent type was found to have no significant association with TVF in patients with known glycemic state, and therefore DES type was not included as potential confounder in the multivariable model. Multivessel disease differed between metabolic groups and was included in the multivariable model, but incomplete revascularization for clinically relevant (based on ischemia detection or invasive measurements) and treatable lesions was not recorded in our database. Nevertheless, in the participating centres, it is common practice to discuss patients with multivessel disease in a Heartteam, aiming at optimal revascularization. Therefore, it may be assumed that there was a low rate of patients with incomplete revascularization of clinically relevant and treatable lesions.

## Conclusions

Not only patients with diabetes but also patients with prediabetes represent a high-risk population. After treatment with new-generation DES, both patient groups had higher risks of ischemic and bleeding events. Differences in major bleeding were mainly attributable to between-group dissimilarities in patient characteristics. Routine assessment of glycemic state may help to identify PCI patients with prediabetes for intensified management of cardiovascular risk factors in order to improve outcome.

## Supplementary Information

**Additional file 1: Table S1. **Use of antiplatelet and oral anticoagulant therapy at 3-year follow-up. **Table S2. **Subgroup analysis of patients with acute coronary syndrome.

## Data Availability

The datasets generated and/or analysed during the current study are not publicly available due to privacy restrictions for pseudo-anonymized data under European law, but can be made available from the corresponding author on reasonable request after signing a data-sharing agreement.

## Abbreviations

ACS: Acute coronary syndrome
BARC: Bleeding Academic Research Consortium
CI: Confidence interval
DAPT: Dual antiplatelet therapy
DES: Drug-eluting stent
FPG: Fasting plasma glucose
HbA1c: Hemoglobin A1c
HR: Hazard ratio
MI: Myocardial infarction
PCI: Percutaneous coronary intervention
TIMI: Thrombolysis in myocardial infarction
TVF: Target vessel failure
