# Supplementary material for: Impact of prediabetes and diabetes on 3-year outcome of patients treated with new-generation drug-eluting stents in two large-scale randomized clinical trials
Source: Cardiovasc Diabetol. 2021 Oct 30;20:217. doi: 10.1186/s12933-021-01405-4 (PMC8557556; doi:10.1186/s12933-021-01405-4)
Supplement: Supplementary file 1 — Additional file 1: Table S1. Use of antiplatelet and oral anticoagulant therapy at 3-year follow-up. Table S2. Subgroup analysis of patients with acute coronary syndrome. [file 12933_2021_1405_MOESM1_ESM.docx]

**Additional file 1.**

**Impact of Prediabetes and Diabetes on 3-Year Outcome of Patients**

**Treated with New-Generation Drug-Eluting Stents in**

**Two Large-Scale Randomized Clinical Trials**

Eline H Ploumen, MD^1,2^; Tineke H Pinxterhuis, MD^1,2^; Paolo Zocca, MD PhD^1^;

Ariel Roguin, MD PhD^3^; Rutger L Anthonio MD^4^; Carl E Schotborgh, MD^5^;

Edouard Benit, MD^6^; Adel Aminian, MD^7^; Peter W Danse, MD PhD^8^;

Carine JM Doggen, PhD^2^; Clemens von Birgelen, MD PhD^1,2^;

Marlies M Kok, MD PhD^1^

^1^ Department of Cardiology, Thoraxcentrum Twente, Medisch Spectrum Twente, Enschede, the Netherlands

^2^ Department of Health Technology and Services Research, Faculty of Behavioural, Management and Social

Sciences, Technical Medical Centre, University of Twente, Enschede, Netherlands

^3^ Department of Cardiology, Hillel Yaffe Medical Center, Hadera and B. Rappaport-Faculty of Medicine, Israel
 Institute of Technology, Haifa, Israel

^4^ Department of Cardiology, Treant Zorggroep, Scheper Hospital, Emmen, Netherlands

^5^ Department of Cardiology, Haga Hospital, The Hague, Netherlands

^6^ Department of Cardiology, Jessa Hospital, Hasselt, Belgium

^7^ Department of Cardiology, Centre Hospitalier Universitaire de Charleroi, Charleroi, Belgium

^8^ Department of Cardiology, Rijnstate Hospital, Arnhem, Netherlands

**Table S1. Use of antiplatelet and oral anticoagulant therapy at 3-year follow-up Page 2.**

**Table S2. Subgroup analysis of patients with acute coronary syndrome Page 3.**

**Table S1. Use of antiplatelet and oral anticoagulant therapy at 3-year follow-up**

|  | **Normoglycemia n = 2,240** | **Prediabetes n = 458** | **Diabetes n = 1,333** | **P-value** |
| --- | --- | --- | --- | --- |
| **Aspirin** | 1,875 (83.7) | 363 (79.3) | 1,071 (80.3) | 0.010 |
| **Dual antiplatelet therapy** | 127 (5.7) | 42 (9.2) | 161 (12.1) | <0.001 |
| **Clopidogrel** | 73 (3.3) | 30 (6.6) | 98 (7.4) | <0.001 |
| **Ticagrelor** | 48 (2.1) | 11 (2.4) | 51 (3.8) | 0.010 |
| **Prasugrel** | 6 (0.3) | 1 (0.2) | 12 (0.9) | 0.020 |
| **Direct oral anticoagulant** | 116 (5.2) | 31 (6.8) | 78 (5.9) | 0.35 |
| **Vitamin K antagonist** | 168 (7.5) | 46 (10.0) | 143 (10.7) | 0.003 |

Values are n(%). Data available in 4,031/4,330(93.1%) patients.

**Table S2. Subgroup analysis of patients with acute coronary syndrome**

|  | **Normoglycemia**  **n=2,353** | **Prediabetes n=489** | **Diabetes**  **n=1,488** | **P-value  prediabetes vs. NG** | **HR (95%CI) prediabetes  vs. NG** | **P-value diabetes  vs. NG** | **HR (95%CI)  diabetes vs. NG** |
| --- | --- | --- | --- | --- | --- | --- | --- |
| **Target vessel failure** | | | | | | | |
| Acute coronary syndrome | 88/1,673 (5.3) | 34/338 (10.1) | 122/1,002 (12.2) | 0.001 | 1.97 (1.32-2.92) | <0.001 | 2.43 (1.84-3.19) |
| Stable angina | 54/680 (7.9) | 20/151 (13.2) | 75/486 (15.4) | 0.038 | 1.72 (1.03-2.88) | <0.001 | 2.04 (1.44-2.89) |
| **Major bleeding** | | | | | | | |
| Acute coronary syndrome | 37/1,673 (2.2) | 17/338 (5.0) | 38/1,002 (3.8) | 0.004 | 2.35 (1.32-4.17) | 0.014 | 1.77 (1.12-2.78) |
| Stable angina | 17/680 (2.5) | 2/151 (1.3) | 21/486 (4.3) | 0.40 | 0.53 (0.12-2.29) | 0.08 | 1.77 (0.93-3.36) |

Values are n/N (%). Formal interaction testing revealed no significant interactions. Abbreviations: NG = normoglycemia.
